# Detecting Demographic Influences on Measures of Spatial Ability with Rasch Tree Analysis

**DOI:** 10.3390/jintelligence14060106

**Published:** 2026-06-11

**Authors:** Justice Dadzie, Christopher A. Ocheni, Daniel O. Oyeniran, Joni M. Lakin

**Affiliations:** Department of Educational Studies, College of Education, The University of Alabama, Tuscaloosa, AL 35487, USA; jdadzie@crimson.ua.edu (J.D.); caocheni@crimson.ua.edu (C.A.O.); dooyeniran@crimson.ua.edu (D.O.O.)

**Keywords:** spatial ability, Rasch model, subgroup differences, differential item functioning (DIF), Rasch tree, STEM education

## Abstract

This study applied Rasch modeling to examine the psychometric properties of two spatial assessments, the Mental Rotation Test (MRT) and Object Assembly (OA), and used group comparisons to explore demographic patterns across the MRT, OA, and Surface Development (SD). Participants were 123 students in Grades 3–8 recruited from university-affiliated summer programs. For the MRT and OA, most items showed acceptable model–data fit, with infit mean square values generally within the expected range of 0.70 to 1.30, although a small number of items displayed evidence of underfit or overfit. Wright maps indicated generally appropriate alignment between item difficulty and participant ability, supporting the usefulness of these measures for distinguishing low-to-moderate levels of spatial skill. Group comparisons based on Rasch-derived theta estimates showed a substantial overlap across gender and race groups on the MRT and OA, with small and non-significant differences. SD raw-score comparisons showed similarly modest subgroup differences. To evaluate measurement invariance at the item level, separate Rasch tree analyses were conducted for the MRT and OA using gender and race as potential splitting variables. Methodologically, the study contributes by combining Rasch-based evaluation of item functioning with exploratory subgroup visualization to examine how spatial assessment performance may vary across demographic groups while maintaining consistency with the psychometric assumptions of the model. Overall, the findings support the value of Rasch-based methods for evaluating the measurement quality of spatial assessments and demonstrate that limited mean group differences at the overall score level can coexist with meaningful item-level noninvariance.

## 1. Introduction

Spatial ability is a fundamental cognitive skill associated with problem solving, visualization, and reasoning across many domains, including science, technology, engineering, and mathematics (STEM; Andersen, 2014; Buckley et al., 2018; Andersen, 2014; Bodzin, 2011; Hawes et al., 2022; Marunić & Glažar, 2014). It allows individuals to mentally manipulate objects, rotate spatial representations, and interpret complex visual information. These abilities are strongly linked to success in mathematics, engineering, and scientific reasoning (Cheng & Mix, 2014; Xie et al., 2020). Because spatial reasoning plays a critical role in STEM learning and career development, researchers have increasingly focused on understanding how spatial abilities are measured and how they vary across individuals and demographic groups (Atit et al., 2022; Froiland & Davison, 2020; Lakin & Wai, 2020).

Many instruments have been developed to measure spatial ability across different contexts (Uttal et al., 2024). Among the most widely used assessments are the Mental Rotation Test (MRT; Shepard & Metzler, 1971; Voyer et al., 1995), Object Assembly (OA; Mitolo et al., 2015; Kell & Lubinski, 2013), and Surface Development (SD; Buckley et al., 2018). These instruments capture distinct but related aspects of spatial reasoning (Hegarty & Waller, 2005; Lohman, 1996; Ramful et al., 2017). The MRT measures the ability to mentally rotate three-dimensional objects, OA evaluates the ability to mentally assemble components into meaningful structures, and SD assesses the ability to transform two-dimensional representations into three-dimensional forms. Together, these measures capture both dynamic and structural components of spatial reasoning (Dehn, 2022; Mix et al., 2016).

Research on spatial ability has documented considerable individual differences in performance as well as variation in the strategies individuals use to solve spatial tasks (Gohm et al., 1998; Hegarty & Waller, 2005; Voyer et al., 2020). Some studies have also suggested that spatial ability development is influenced by environmental exposure and learning opportunities, including engagement in spatially rich activities such as drawing, construction, or design tasks (Uttal et al., 2013; Yang et al., 2020). Another line of research focuses on how gendered differences in spatially rich activities might in fact lead to commonly observed sex differences in spatial task performance (Baenninger & Newcombe, 1989; Coyle, 2022; Heo & Toomey, 2020). These findings highlight the importance of ensuring that spatial ability assessments measure the intended construct accurately and fairly across diverse populations.

Psychometric modeling approaches are commonly used to evaluate the quality of measurement instruments and to examine how test items function across individuals. One widely used framework is the Rasch model, which estimates item difficulty and person ability on a shared logit scale while providing diagnostic information about item functioning (Doyle et al., 2012; Bond & Fox, 2015; Boone et al., 2014). Rasch modeling allows researchers to assess whether items align with the expectations of a measurement model and whether they effectively differentiate individuals across levels of ability.

A central assumption of Rasch measurement is parameter invariance, meaning that item difficulty should remain stable across groups when individuals possess the same level of the latent trait. Violations of this assumption may indicate differential item functioning (DIF), which occurs when individuals from different demographic groups with equivalent ability have different probabilities of responding correctly to a particular item (Zwick, 2025). When DIF is present, observed group differences may reflect measurement artifacts rather than true differences in ability (Bond & Fox, 2015; Boone et al., 2014; Atit et al., 2020). Examining differential item functioning (DIF) therefore provides an important way to determine whether spatial ability measures operate equitably across participants and whether specific item features may contribute to subgroup differences in performance (Rajeb et al., 2024).

In spatial ability research, examining DIF is particularly important because item performance may be influenced by differences in experience, strategy use, or familiarity with spatial tasks (Uttal et al., 2013; Hegarty, 2018). Additionally, item characteristics such as visual complexity, object orientation, and transformation demands may influence how individuals approach spatial reasoning problems (Rajeb et al., 2024; Shi et al., 2023). Identifying DIF can therefore provide valuable insight into how specific item features interact with participant characteristics and influence spatial test performance.

Traditional approaches to DIF detection typically rely on predefined group comparisons, such as evaluating differences across gender or racial groups (Zwick, 2025). While these methods can identify straightforward group differences, they may overlook more complex patterns of measurement noninvariance that arise when multiple demographic variables interact. As a result, researchers have increasingly explored data-driven methods that allow subgroup differences in item functioning to emerge directly from the data.

One such approach is Rasch tree analysis, which combines the Rasch measurement model with recursive partitioning techniques to identify subgroups in which item parameters may differ significantly (Strobl et al., 2015). Rather than requiring researchers to specify group comparisons entirely in advance, Rasch tree models can evaluate covariates such as gender and race to examine whether item parameters remain stable across subgroups. When parameter instability is detected, the sample may be partitioned into subgroups and separate Rasch models estimated within each node. This approach offers a useful framework for exploring potential measurement noninvariance because it links subgroup differentiation directly to item functioning. In the present study, this perspective informed the examination of subgroup patterns for the Mental Rotation Test (MRT) and Object Assembly (OA), two measures with clearly dichotomous item structures appropriate for Rasch-based estimation. This focus helps maintain consistency between the substantive interest in demographic variation and the psychometric requirements of the measurement model (Strobl et al., 2015).

Recent research has demonstrated the usefulness of Rasch tree models for detecting subgroup differences in cognitive and educational assessments (Krist et al., 2025). By identifying subgroup-specific patterns of item difficulty, this approach helps researchers evaluate whether measurement instruments operate equivalently across diverse populations.

Despite the growing literature on spatial ability measurement, an important gap remains in how subgroup differences are examined alongside psychometric evidence about item functioning. Much of the prior work has focused either on overall group differences in performance or on broader discussions of fairness, while fewer studies have combined Rasch-based evaluation of item quality with subgroup-oriented analysis in a way that directly informs the interpretation of spatial assessments. Recent studies have advanced understanding of item characteristics, assessment challenges, and subgroup-sensitive psychometric methods, but these strands of work are not always integrated within the same empirical study, particularly in research involving school-age learners (Krist et al., 2025; Uttal et al., 2024). The present study addresses this gap by examining item functioning and demographic performance patterns together while maintaining consistency between the psychometric framework and the structure of the measures being analyzed.

Building on this work, the present study integrates spatial ability assessment within a Rasch-based psychometric framework to evaluate measurement quality and demographic influences on performance. Rasch analysis was used to estimate item difficulty, person ability, and model fit for the Mental Rotation Test (MRT) and Object Assembly (OA). Surface Development (SD) was retained as a complementary spatial measure for descriptive and group-comparison purposes because it represents an important aspect of spatial reasoning, but it was not treated as part of the Rasch-based analyses. This distinction helps maintain consistency between the study’s substantive interest in multiple dimensions of spatial ability and the psychometric requirements of Rasch-based estimation (Bond & Fox, 2015; Boone et al., 2014; Effatpanah et al., 2025). Specifically, this study addresses three research questions: (1) To what extent do the MRT and OA items fit a Rasch model? (2) Are there mean differences in spatial ability across demographic groups? and (3) What subgroup patterns emerge in exploratory score-based comparisons of combined MRT and OA performance across gender and race?

## 2. Materials and Methods

A Rasch modeling approach was applied to estimate item difficulty, person ability, and model fit statistics for assessing spatial ability and demographic effects. The Rasch model predicted the probability that a person with a given ability level would correctly answer a particular item, based on the difference between person ability and item difficulty on a shared logit scale.

The model is expressed as

$$P(X_{ni}=1)=\frac{e^{\left(\theta_{n}-\beta_{i}\right)}}{1+e^{\left(\theta_{n}-\beta_{i}\right)}}$$

where $P(X_{ni}=1)$ represents the probability that person n answers item i correctly, $\theta_{n}$ denotes the person’s ability, and $\beta_{i}$ represents the difficulty of the item. The log-odds of a correct response increase linearly with the difference between a person’s ability and the item’s difficulty.

Item and person parameters were estimated using conditional maximum likelihood estimation. Model–data fit was evaluated using infit and outfit mean square (MSQ) statistics, with acceptable fit values ranging between 0.7 and 1.3. Items outside these bounds were flagged as potential misfits and further examined to ensure construct validity.

To identify subgroup differences and detect potential measurement bias, Rasch tree analysis was conducted using recursive partitioning (Strobl et al., 2015). This method combined the Rasch model with a decision-tree algorithm to identify subgroups of participants exhibiting different parameter estimates based on demographic covariates such as gender and race.

The Rasch tree can be expressed as

$$P(X_{ni}=1|\theta_{n},\beta_{i},Z_{n})=\frac{e^{\left(\theta_{n,g}-\beta_{i,g}\right)}}{1+e^{\left(\theta_{n,g}-\beta_{i,g}\right)}},\;g=1,2,\ldots ,G$$

where $Z_{n}$ is a vector of person covariates used for splitting (e.g., gender, race), and g represents the subgroup (terminal node) identified by the recursive partitioning algorithm. Each subgroup g received its own Rasch model, with parameters $\theta_{n,g}$ and $\beta_{i,g}$ estimated independently within that node.

Likelihood ratio (LR) tests were used to evaluate parameter instability across covariates. When significant instability was detected (*p* < .05), the algorithm split the data at the covariate producing the largest LR statistic. This recursive process continued until no further significant splits were found. The resulting tree provided a data-driven representation of differential item functioning (DIF) and subgroup-specific performance patterns, thereby enhancing fairness and interpretability in spatial ability measurement.

### 2.1. Participants

A total of 123 students from grades 3 through 8 participated in the study. Participants were recruited through summer camp programs affiliated with universities in the Southeastern and Midwestern United States. Recruitment involved collaboration with camps that offered diverse educational, artistic, and STEM experiences, ensuring access to a broad participant pool. Parents or guardians were invited via email to register their children and provide demographic information, including age, gender, and race. Each participant received a gift card incentive upon completing the assessments. The final sample included 75 males (61%) and 48 females (39%). Participants identified racially as 73% White (*n* = 90), 12% Asian (*n* = 15), 6.5% Native American (*n* = 8), 3.3% Black or African American (*n* = 4), 1.6% Hispanic (*n* = 2), and 3.3% Other (*n* = 4).

Although the sample was adequate for the planned analyses, some racial subgroups were small and unevenly distributed across categories, which warrants caution when interpreting subgroup-specific findings, particularly those derived from recursive partitioning analyses.

Three spatial ability assessments were used in this study: the Mental Rotation Test (MRT), Object Assembly (OA), and Surface Development (SD). The MRT consisted of 17 items adapted from Shepard and Metzler’s (1971) and Vandenberg and Kuse’s (1978) mental rotation paradigms to measure dynamic aspects of spatial visualization. The OA comprised 15 tasks designed to assess spatial organization and part–whole integration skills, reflecting participants’ ability to mentally assemble or disassemble objects. The SD included 17 items that measured participants’ ability to mentally transform two-dimensional representations into three-dimensional forms, evaluating the static and structural components of spatial reasoning. All tasks were pilot-tested with both middle school and college-aged participants to ensure item clarity, age appropriateness, and internal consistency before the main study. Feedback from pilot testing informed refinements in item layout and instructions. 

### 2.2. Procedure

All spatial ability assessments were administered online during scheduled summer camp sessions held in classroom environments. Participants completed the Mental Rotation Test (MRT), Object Assembly (OA), and Surface Development (SD) tasks individually under standardized testing conditions. Trained research assistants provided uniform instructions and ensured a quiet, controlled testing environment to minimize distractions and maintain test integrity.

Each assessment session lasted approximately 30 min, allowing participants sufficient time to complete all three spatial ability tasks. The order of test administration was counterbalanced across sessions to reduce potential order effects. Participants were instructed to work carefully and to attempt all items within the allotted time. Participant anonymity was maintained through the use of coded identifiers.

### 2.3. Data Analysis

Comprehensive psychometric analyses were conducted to evaluate the measurement quality of the spatial ability assessments and to examine the influence of demographic variables such as gender and race. Internal consistency reliability for each task was assessed using corrected item–total correlations and Cronbach’s alpha to ensure adequate scale stability.

To analyze item difficulty and measurement precision, Rasch modeling was applied to the Mental Rotation Test (MRT) and Object Assembly (OA) using the eRm package in R 4.5.1. These two instruments were selected because they consist of discrete, dichotomously scored items with clearly defined correct and incorrect responses, making them appropriate for Rasch model estimation and interpretation (Bond & Fox, 2015; Boone et al., 2014). In contrast, Surface Development (SD) was retained as a complementary spatial measure for descriptive comparison only. Although SD captures an important aspect of spatial reasoning, its task structure, comprising multiple questions for each stimuli, was not appropriate for the item-level dichotomous framework used in the present Rasch analyses. For this reason, SD was not included in the Rasch-based modeling and was instead interpreted descriptively to maintain consistency between the psychometric method and the structure of the measure.

Item and person parameters were estimated using conditional maximum likelihood estimation. Fit statistics, including infit and outfit mean square (MSQ) values, were examined to evaluate model–data alignment, with acceptable values ranging from 0.7 to 1.3. Items outside this range were identified as potential misfits and reviewed for construct validity. Person–item Wright maps were generated for both the MRT and OA to visualize the relationship between participants’ ability levels and item difficulties on a common logit scale.

To examine demographic influences on item functioning, Rasch tree analysis was applied to the Mental Rotation Test (MRT) and Object Assembly (OA). The Rasch tree procedure evaluated parameter instability across gender and race using likelihood ratio tests and recursively partitioned the sample only when the splitting criterion met the pre-specified significance threshold. This analytic approach made it possible to examine whether item difficulty estimates remained stable across demographic subgroups while maintaining consistency with the assumptions of the measurement model (Strobl et al., 2015). Given the modest sample size and uneven racial subgroup distributions, race was collapsed into two groups (white and non-white), and the Rasch tree findings were interpreted as exploratory and evaluated with particular caution. Decision tree visualizations were generated to summarize subgroup differentiation identified through the Rasch tree models.

Additionally, independent-samples Welch’s *t*-tests were conducted to examine both gender and race differences across the three assessments (MRT, OA, and SD). For the MRT and OA, these comparisons were based on Rasch-derived theta estimates, whereas for SD they were based on raw scores. These analyses were used to describe mean-level performance patterns across measures, whereas Rasch-based analyses were reserved for the MRT and OA. 

This analytical framework provided a data-driven understanding of how spatial ability measures functioned across demographic groups while preserving consistency between the study’s substantive aims and its psychometric methods. By focusing Rasch and Rasch tree analyses on the MRT and OA, the study prioritized methodological alignment in the estimation of item difficulty, model fit, and subgroup-related parameter instability. At the same time, the inclusion of SD in descriptive group comparisons allowed the study to retain broader coverage of spatial reasoning without overstating its compatibility with Rasch-based item analysis.

No generative artificial intelligence (GenAI) tools were used to generate ideas, analysis, or substantive content of this manuscript. AI-assisted tools were used only for minor language editing, including grammar, spelling, punctuation, and formatting.

## 3. Results

The results are organized in two parts. The first part presents the Rasch model findings related to item difficulty and model fit for the Mental Rotation Test (MRT) and Object Assembly (OA). The second part reports demographic analyses, including gender and racial group differences across the MRT, OA, and Surface Development (SD) tasks.

### 3.1. Item Difficulty and Model Fit

**Figure 1:**
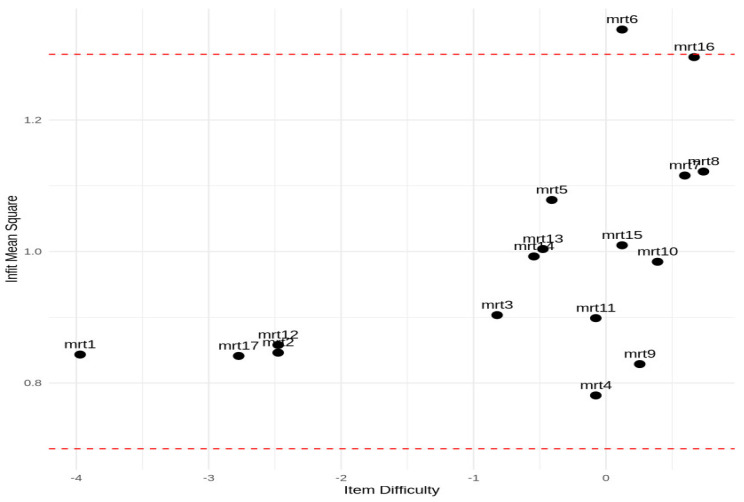
Item fit plot for the Mental Rotation Test (MRT).

**Figure 2:**
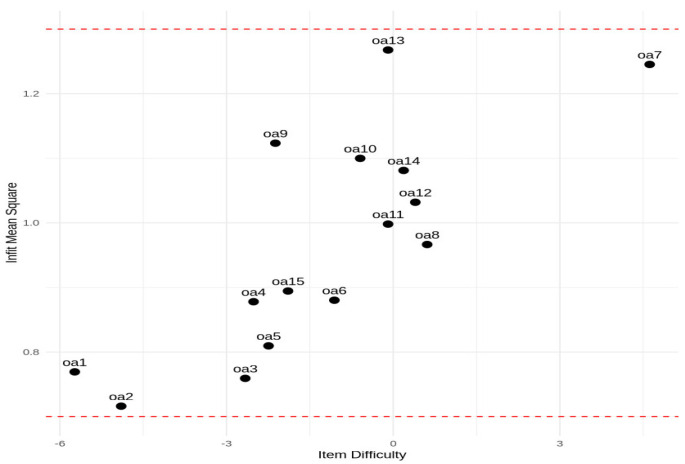
Item fit plot for the Object Assembly (OA).

**Figure 3:**
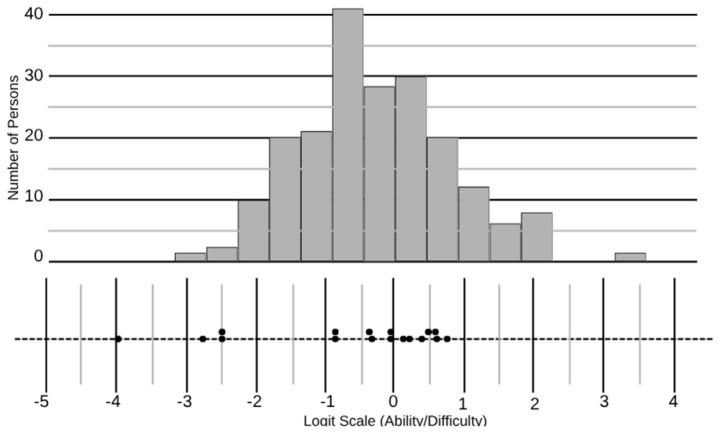
Wright person-item map for the Mental Rotation Test (MRT).

**Figure 4:**
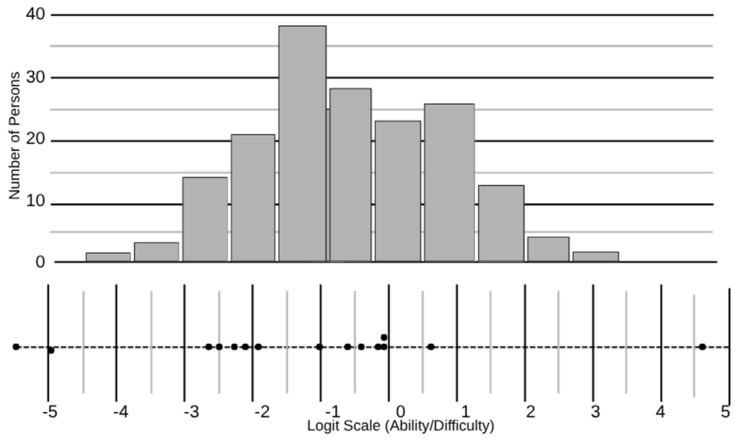
Wright person-item map for the Object Assembly (OA).

Most items showed acceptable model–data fit, clustering near the expected infit MSQ of 1.0 and remaining within the dashed acceptable band (≈0.70–1.30). For the MRT (Figure 1), item difficulties ranged from very easy to moderately hard, and fit was generally stable; however, mrt6 and mrt16 exceeded the upper infit boundary, indicating underfit, while mrt4 fell below the lower boundary, suggesting possible overfit. The underfit observed for mrt6 and mrt16 may reflect additional cognitive demands associated with more complex rotations, less transparent visual alignment, or the use of multiple solution strategies across participants. By contrast, the overfit for mrt4 may indicate that the item functioned too predictably relative to the underlying latent trait. For the OA (Figure 2), item difficulties spanned a broader range, and most items fit the Rasch model well. However, oa13 and oa7 approached or exceeded the upper fit boundary, suggesting that these items may have involved added visual complexity or multidimensional demands related to part-whole integration. Overall, the Wright maps for the MRT and OA indicated generally appropriate targeting of item difficulty to participant ability, supporting the usefulness of both measures for distinguishing low-to-moderate levels of spatial skill (Bond & Fox, 2015; Buckley et al., 2018). See Figure 3 and Figure 4, respectively.

Each point represents an MRT item. Items within the 0.7–1.3 infit range indicate acceptable fit; deviations suggest potential multidimensionality or misfit.

### 3.2. Demographic Differences in Spatial Ability

**Figure 5:**
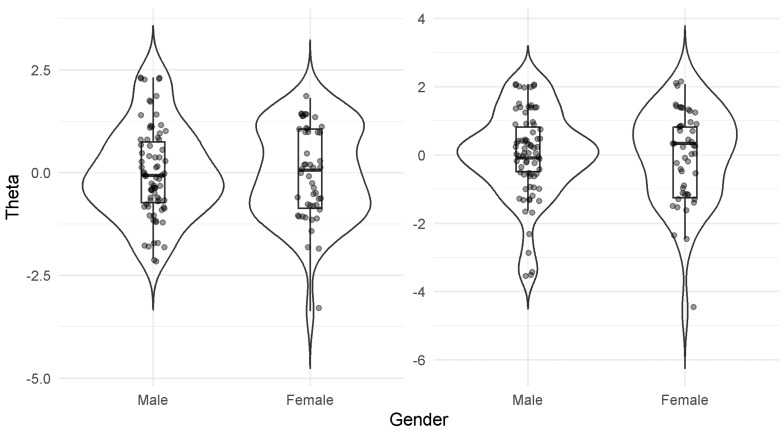
Distributions of Rasch (1PL) EAP Theta Scores by Gender for the Mental Rotation Test (MRT—Left) and Object Assembly (OA—Right).

**Figure 6:**
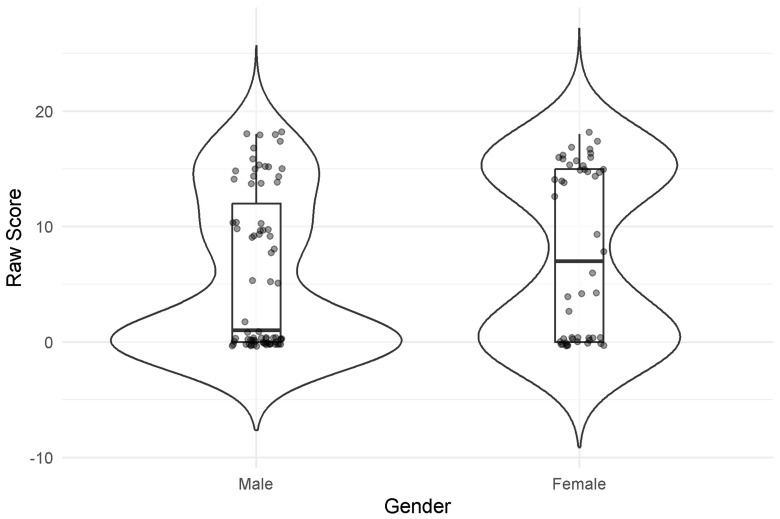
Distribution of Surface Development (SD) Raw Scores by Gender.

**Figure 7:**
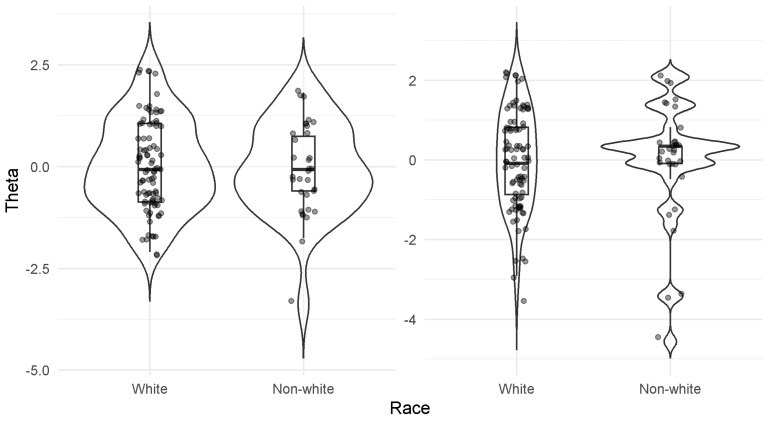
Distributions of Rasch (1PL) EAP Theta Scores by Race for the Mental Rotation Test (MRT—Left) and Object Assembly (OA—Right).

**Figure 8:**
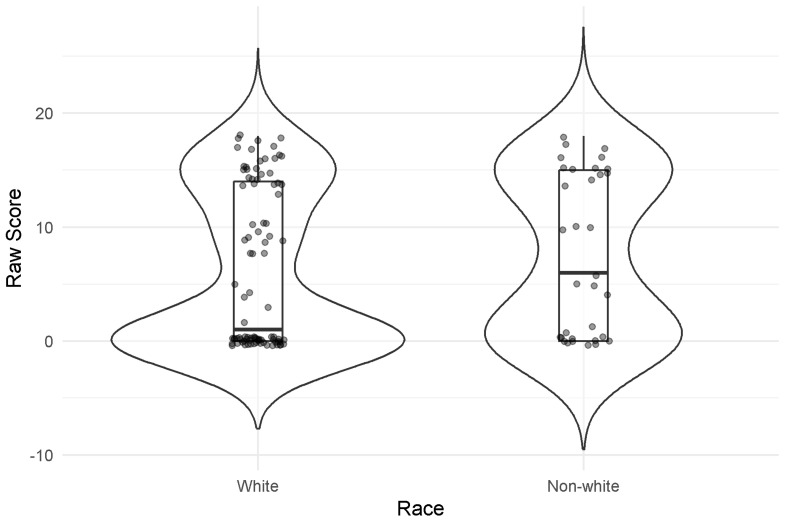
Distribution of Surface Development Raw Scores by Race.

To examine whether spatial ability differed by gender and race, group comparisons were conducted using Rasch (1PL) EAP theta estimates for the Mental Rotation Test (MRT) and Object Assembly (OA), and raw scores for the Surface Development (SD) task. Because group variances were not assumed to be equal, Welch’s independent-samples *t* tests were used. In addition to significance testing, 95% confidence intervals and Cohen’s d values were reported to provide point estimates and effect size information. Figure 5, Figure 6, Figure 7 and Figure 8 visually display the score distributions, while Table 1 and Table 2 summarize the statistical results. 

Figure 5 and Figure 6 present the distributions of MRT theta, OA theta, and SD raw scores by gender. As shown in Figure 5, the MRT and OA violin plots indicate substantial overlap between male and female participants. The embedded boxplots show similar medians and interquartile ranges, and the spread of individual points suggests broadly comparable performance across the two groups. This visual pattern is consistent with the inferential results presented in Table 1.

Both MRT and OA theta scores showed trivial and non-significant differences between males and females. Gender differences on the Surface Development task are shown in Figure 6. The plot suggests that female participants tended to score somewhat higher than male participants, although there was still considerable overlap between the two groups. As reported in Table 1, males obtained a mean raw score of 5.83 (*SD* = 6.72, *n* = 75), whereas females obtained a mean raw score of 7.85 (*SD* = 7.34, *n* = 48). However, this difference did not reach statistical significance in this sample (*t*(93.82) = −1.54, *p* = .13).

Taken together, the gender analyses indicate that males and females performed very similarly on the MRT and OA, with virtually no difference in latent ability estimates. For SD, females showed a modest numerical advantage, but the difference remained non-significant. The figures also suggest somewhat wider score dispersion among females, especially for SD, but the overlap between groups remained substantial. Overall, these findings do not support strong gender-based differences in overall spatial performance in this sample.

Figure 7 and Figure 8 display score distributions by race, comparing White and Non-white participants. As shown in Figure 7, the MRT and OA theta distributions overlap heavily across racial groups, with similar medians and considerable within-group variability. This visual pattern again matches the inferential findings summarized in Table 2. We found that none of the mean differences for race were significant, although SD again showed larger differences.

Overall, the race comparisons indicate minimal differences between White and Non-white participants across the MRT, OA, and SD. The very small effect sizes for the MRT and OA suggest that race contributed little to differences in latent ability on those measures. Although Non-white participants showed somewhat higher mean SD raw scores, the confidence interval included zero and the effect remained small. The violin plots reinforce this interpretation by showing broad overlap in score distributions across groups.

Across both gender and race analyses, the results consistently showed that group differences were small and statistically non-significant. For the MRT and OA, mean differences were close to zero and Cohen’s *d* values indicated negligible effects. For SD, females and Non-white participants showed somewhat higher raw-score averages than their comparison groups, but these differences were still small and not statistically reliable. The broad overlap visible in Figure 5, Figure 6, Figure 7 and Figure 8 further suggests that within-group variability was greater than between-group differences. 

### 3.3. Rasch Tree Analysis and Item-Level DIF 

**Figure 9:**
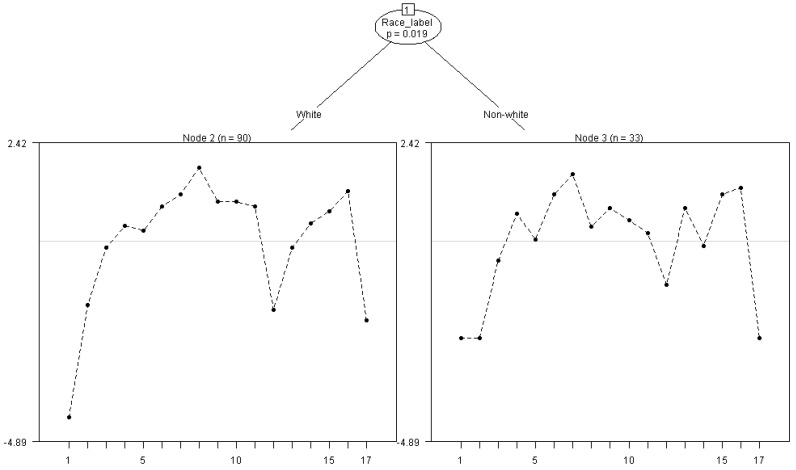
Rasch Tree Analysis of Demographic Subgroup Differences in Mental Rotation Test (MRT) Item Functioning. Each point indicates the level of differential functioning by item.

**Figure 10:**
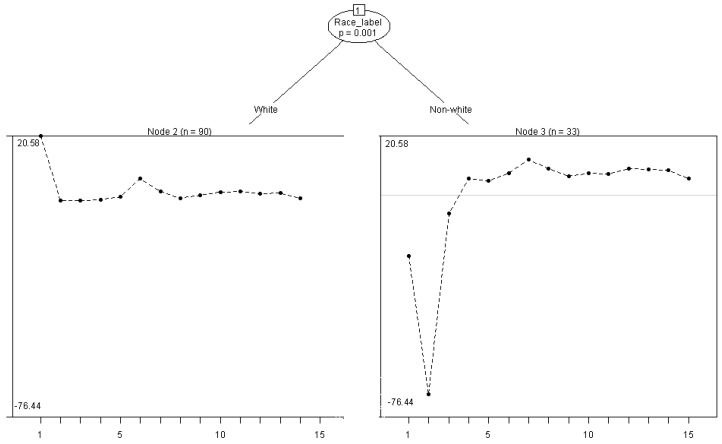
Rasch Tree Analysis of Demographic Subgroup Differences in Object Assembly (OA) Item Functioning. Each point indicates the level of differential functioning by item.

To examine whether item difficulty parameters remained invariant across demographic groups, separate Rasch tree analyses were conducted for the MRT and OA using gender and race as potential splitting variables. The resulting trees are presented in Figure 9 and Figure 10.

Figure 9 presents the Rasch tree for the MRT. The analysis identified a significant split on race, *p* = .019, separating White participants (*n* = 90) from Non-white participants (*n* = 33). No additional split on gender emerged after this division. This result indicates that the MRT item difficulty profile differed sufficiently by race to trigger subgroup partitioning, suggesting the presence of race-related differential item functioning at the item level.

Figure 10 presents the Rasch tree for OA. Here again, the first and only significant split occurred on race, with even stronger evidence of parameter instability than in the MRT, *p* = .001. The sample was divided into the same White (*n* = 90) and Non-white (*n* = 33) subgroups, and no further split on gender was observed. This pattern indicates that race-related item-level noninvariance was more pronounced for OA than for the MRT in the present sample.

To clarify which items contributed most strongly to these subgroup differences, ranked DIF magnitude plots were generated separately for race and gender. These plots show the absolute difference in Rasch item difficulty across groups, with larger values indicating stronger DIF. A dashed vertical line marks an absolute DIF threshold of 0.50, which was used as a practical indicator of notable DIF.

**Figure 11:**
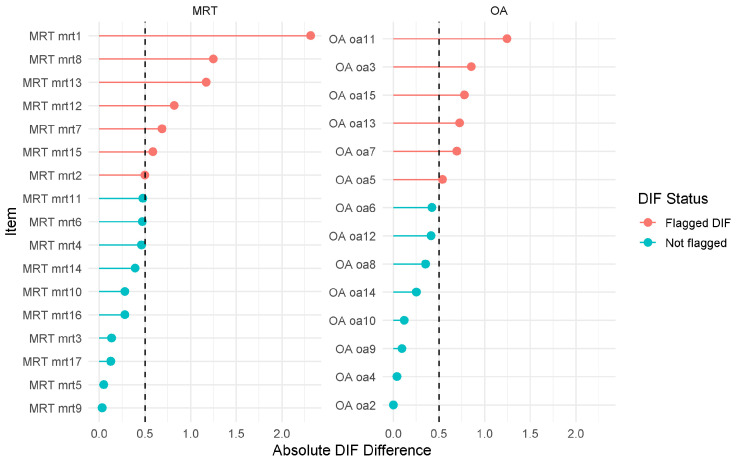
Ranked DIF Magnitude by Race Across MRT and OA.

The race-based DIF plot in Figure 11 showed that several MRT and OA items exceeded the 0.50 practical DIF threshold. For the MRT, the largest race-related DIF was observed for mrt1, followed by mrt8, mrt13, mrt12, mrt7, and mrt15. Among these, mrt1 displayed the strongest subgroup discrepancy, with an absolute DIF magnitude well above the threshold. For OA, the largest race-related DIF was observed for oa11, followed by oa3, oa15, oa13, and oa7. These results suggest that OA showed not only a stronger Rasch tree split by race, but also several individual items with substantial subgroup-related difficulty shifts. In contrast, other items in both the MRT and OA fell below the threshold and appeared to contribute less to race-based noninvariance.

**Figure 12:**
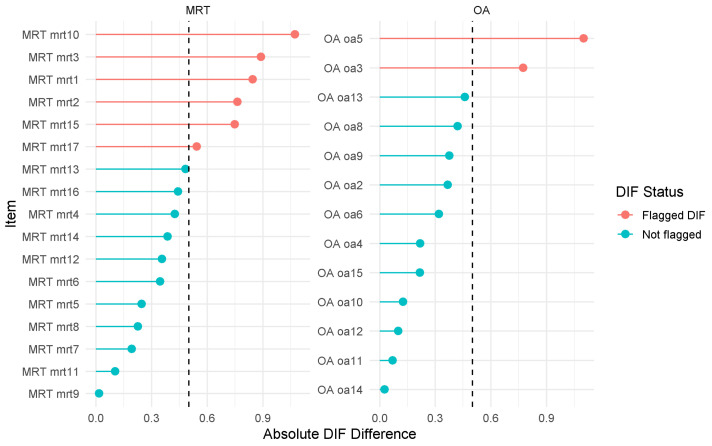
Ranked DIF Magnitude by Gender Across MRT and OA.

The gender-based DIF plot in Figure 12 revealed a smaller but still meaningful pattern. For the MRT, the most prominent gender DIF items were mrt10, mrt3, mrt1, mrt2, mrt15, and mrt17, several of which exceeded the 0.50 threshold. For OA, the strongest gender DIF was observed for oa5 and oa3, both of which exceeded the threshold, whereas most remaining OA items fell below it. This pattern suggests that gender-related DIF was present for selected items, but it did not produce a sufficiently strong global instability pattern to emerge as a Rasch tree split in either instrument.

Taken together, the Rasch tree and ranked DIF results suggest that race was the more prominent source of item-level noninvariance in the present dataset. Although overall mean differences by race were small, item-specific difficulty estimates varied enough to produce statistically significant subgroup splits in both the MRT and OA. This finding is important because it shows that the absence of mean group differences does not guarantee item-level invariance. In other words, two groups may appear similar in their average performance while still responding differently to particular items. The OA results were especially notable in this regard, as both the Rasch tree and the ranked DIF plot indicated stronger race-related DIF for OA than for the MRT (Effatpanah et al., 2025; Strobl et al., 2015).

## 4. Discussion

This study examined spatial ability using a Rasch-based psychometric framework to evaluate item functioning, model fit, and demographic patterns in performance. The findings showed that the Mental Rotation Test (MRT) and Object Assembly (OA) generally fit the Rasch model well and demonstrated reasonable alignment between item difficulty and participant ability, supporting the use of both measures for interpreting item difficulty and person ability on a common logit scale (Bond & Fox, 2015; Boone et al., 2014). Beyond overall model fit, the study compared demographic patterns using Rasch theta estimates for the MRT and OA and raw scores for Surface Development (SD). To assess whether item functioning remained stable across groups, separate Rasch tree analyses were conducted for the MRT and OA, followed by item-level differential item functioning analyses. This combined approach made it possible to examine demographic differences not only at the level of overall scores but also at the level of individual items, which is critical for evaluating fairness and measurement invariance in spatial assessments (Strobl et al., 2015; Krist et al., 2025).

### 4.1. Item Difficulty and Model Fit 

The Rasch analysis revealed that both the MRT and OA demonstrated acceptable psychometric quality, with most items fitting the model expectations. A small number of items in each test showed misfit, suggesting multidimensionality, lower discrimination, or unexpected response patterns. These findings align with previous studies reporting that spatial ability measures often capture overlapping but distinct cognitive processes such as rotation, visualization, and spatial structuring (Buckley et al., 2018; Uttal et al., 2024). The OA showed a wider difficulty range than the MRT, implying that the task captured more diverse cognitive demands, including pattern construction and mental synthesis. Similar observations have been made by Carroll (1993) and Linn and Petersen (1985), who noted that spatial reasoning encompasses multiple subskills that may not load onto a single dimension. The Wright maps confirmed adequate targeting between item difficulty and participant ability levels, demonstrating that both instruments effectively measured a broad spectrum of spatial skills.

### 4.2. Gender and Race Difference in Spatial Ability

Gender- and race-related differences in spatial ability were modest at the overall score level. On the Rasch/EAP latent scale, the MRT and OA showed substantial overlap across both gender and race groups, indicating limited separation in average latent performance. Males and females did not differ significantly on the MRT or OA, and White and Non-white participants also showed only minimal differences on these two measures. This pattern does not support a simple subgroup-gap interpretation. Instead, it is more consistent with scholarship showing that spatial performance varies according to task demands, opportunities for practice, and prior learning experiences rather than reflecting a fixed or uniform demographic difference (Kell & Lubinski, 2013; Uttal et al., 2013; Yang et al., 2020). It is also in line with evidence that spatial skills can be strengthened through targeted exposure, training, and instructional support (Nazareth et al., 2019; Sorby et al., 2022).

For Surface Development, the comparisons were based on raw scores rather than Rasch theta estimates. Although females scored somewhat higher than males and Non-white participants scored somewhat higher than White participants, neither difference reached statistical significance. These SD patterns therefore point to modest descriptive variation rather than meaningful subgroup separation. Such a result is consistent with literature suggesting that demographic patterns in spatial ability are not uniform across measures, but instead vary by task type, response demands, and prior experience (Hegarty, 2018; Voyer et al., 2020). It also aligns with evidence that spatial skills are shaped by exposure, instruction, and practice, which can weaken or complicate simple demographic comparisons (Kell & Lubinski, 2013; Uttal et al., 2013; Yang et al., 2020). Accordingly, the present SD findings should be interpreted cautiously as small, non-significant differences rather than evidence of robust demographic gaps in spatial performance.

However, the item-level analyses revealed a more complex pattern than the overall score comparisons alone would suggest. Although group mean differences were small, the Rasch tree analyses for both the MRT and OA identified significant splits by race, and the ranked DIF plots showed that several specific items displayed notable subgroup-related difficulty differences. Gender-related DIF was also evident for selected items, although it was not strong enough to produce a Rasch tree split in either instrument. This result aligns with established psychometric work showing that measurement noninvariance can emerge at the item level even when total-score or latent mean differences remain small (Bond & Fox, 2015; Boone et al., 2014). Rasch tree methodology was developed precisely to detect these subgroup-specific instabilities in item parameters, and prior work has shown that such splits can reveal meaningful DIF patterns that would be missed by relying on overall score comparisons alone (Strobl et al., 2015; Krist et al., 2025). From this perspective, the present findings suggest that demographic differences in spatial assessment were not primarily expressed through broad mean differences but through the functioning of particular items. This underscores the importance of integrating score-based and item-level approaches when evaluating fairness in spatial ability measures. 

## 5. Conclusions

Overall, the present study demonstrates that Rasch modeling provides a robust psychometric framework for evaluating spatial ability measures. By applying the Rasch model to the Mental Rotation Test (MRT) and Object Assembly (OA), the study was able to examine item functioning, assess model–data fit, and evaluate the extent to which item difficulty was appropriately aligned with participant ability. The results indicate that both instruments generally exhibited acceptable measurement properties, supporting their use as indicators of spatial reasoning within this sample.

At the same time, the findings make clear that a satisfactory overall model fit does not, by itself, guarantee complete measurement equivalence across demographic groups. Although gender- and race-based comparisons at the overall score level were small and statistically non-significant, the Rasch tree and item-level DIF analyses revealed meaningful subgroup-related differences in the functioning of particular items, especially with respect to race. This pattern underscores an important measurement principle: demographic similarity in average performance can coexist with item-level noninvariance, such that groups with comparable overall scores may nonetheless encounter different levels of difficulty on specific items.

Taken together, these findings highlight the value of integrating overall score comparisons with item-level invariance diagnostics when evaluating spatial assessments. In substantive terms, the results suggest that spatial ability should not be interpreted as a uniform construct expressed identically across all measures and groups. Rather, its assessment is shaped by the interaction of task characteristics, subgroup-related response patterns, and the psychometric behavior of individual items. Accordingly, the study contributes to ongoing efforts to develop spatial measures that are not only reliable and valid but also fair, interpretable, and sensitive to the diverse ways in which spatial reasoning may be expressed across learners.

### 5.1. Implications for Assessment and Education

The findings carry important implications for both spatial assessment and STEM education. First, they demonstrate the value of Rasch modeling as a diagnostic framework for evaluating item quality, model–data fit, and the extent to which item difficulty is appropriately matched to participant ability. By identifying items that deviate from model expectations or display subgroup-related instability, Rasch-based analyses enable researchers and test developers to refine spatial measures in ways that strengthen their psychometric quality, interpretability, and fairness.

Second, although the subgroup patterns observed in the present study were generally modest and should be interpreted with caution, they nonetheless underscore the importance of examining how spatial assessments function across diverse groups of learners. Even when overall mean differences are limited, item-level analyses may reveal patterns of noninvariance that warrant closer scrutiny through item review, fairness evaluation, or additional psychometric investigation. This is particularly important in the assessment of spatial reasoning, given that performance in this domain is shaped not only by underlying ability, but also by variation in learning opportunities, practice, and familiarity with task demands.

From an educational perspective, these findings reinforce the need for spatial assessment and instruction to be designed in ways that are both inclusive and developmentally responsive. If spatial reasoning is influenced by experience and exposure, then assessment practices should avoid treating it as a static or uniformly expressed trait. Instead, measures should be developed and evaluated with attention to the diverse contexts in which learners acquire and demonstrate spatial skills. In this sense, the present study supports ongoing efforts to develop spatial measures that are psychometrically sound, educationally meaningful, and better suited to informing teaching and learning in STEM-related contexts (Cheng & Mix, 2014; Hawes et al., 2022; Sorby, 2009; Yang et al., 2020).

### 5.2. Limitations and Future Directions

Several limitations should be acknowledged. First, although the sample was adequate for the primary Rasch analyses, it was modest for subgroup interpretation and related exploratory comparisons. Accordingly, the study may have had limited sensitivity to detect smaller subgroup differences or more subtle forms of parameter instability, and null or modest findings should therefore be interpreted with appropriate caution. In particular, the racial subgroups were small and unevenly distributed, which required the collapsing of race into two categories. In addition, demographic information was based on parent- or self-reported identification, which may introduce some measurement imprecision in subgroup classification. The use of broader subgroup contrasts may also obscure important heterogeneity within smaller categories. Second, the study did not treat all three spatial measures within the same psychometric framework. Rasch modeling was applied to the Mental Rotation Test (MRT) and Object Assembly (OA), whereas Surface Development (SD) was retained only for descriptive comparison. As a result, the strength of the psychometric evidence is not uniform across all measures. Third, the exploratory decision tree of combined MRT and OA scores was descriptive rather than a formal item-level Rasch tree test of differential item functioning, and it should therefore not be interpreted as definitive evidence of measurement noninvariance. Finally, because the data were cross-sectional and drawn from university-affiliated summer programs, the findings should not be generalized too broadly and do not support causal claims about the development of spatial ability. Future studies should use larger and more diverse samples and apply consistent item-level methods across measures to examine subgroup patterns with greater precision.

## Figures and Tables

**Table 1:** Gender Differences in MRT Theta, OA Theta, and SD Raw Scores.

Measure	Male (*n* = 75) *M* (*SD*)	Female (*n* = 48) *M* (*SD*)	95% CI	*t*	*df*	*p*	*d*
MRT theta	0.02 (1.12)	−0.03 (1.13)	[−0.37, 0.46]	0.21	99.47	.83	0.04
OA theta	0.02 (1.29)	−0.03 (1.37)	[−0.44, 0.54]	0.21	95.90	.83	0.04
SD raw score	5.83 (6.72)	7.85 (7.34)	[−4.64, 0.58]	−1.54	93.82	.13	−0.29

Note. MRT = Mental Rotation Test; OA = Object Assembly; SD = Surface Development. MRT and OA are Rasch (1PL) EAP theta estimates. SD values are raw scores. Welch’s independent-samples *t* test was used.

**Table 2:** Racial Group Differences in MRT and OA IRT Ability Estimates and SD Raw Scores.

	Group Means (SD)						
Measure	White (*n* = 90)	Non-White (*n* = 33)	95% CI	*t*	*df*	*p*	*d*
MRT theta	0.03 (1.11)	−0.07 (1.14)	[−0.37, 0.56]	0.42	56.01	.68	0.09
OA theta	0.01 (1.25)	−0.01 (1.51)	[−0.57, 0.61]	0.05	48.87	.96	0.01
SD raw score	6.22 (6.98)	7.70 (7.08)	[−4.35, 1.40]	−1.03	56.30	.31	−0.21

Note. MRT = Mental Rotation Test; OA = Object Assembly; SD = Surface Development. MRT and OA are Rasch (1PL) EAP theta estimates. SD values are raw scores. Welch’s independent-samples *t* test was used.

## Data Availability

The datasets presented in this article are not readily available because of on-going analysis and grant objectives. Requests to access the datasets should be directed to jlakin@ua.edu.

## Abbreviations

The following abbreviations are used in this manuscript:

MRT	Mental Rotation Test
OA	Object Assembly
SD	Surface Development
DIF	Differential Item Functioning
MSQ	Mean Squared
STEM	Science, Technology, Engineering, and Mathematics
LR	Likelihood Ratio
ANOVA	Analysis of Variance
1PL	1-Parameter Logistic
EAP	Expected Apriori
